# Serum 25(OH)D levels are associated with disease activity and renal involvement in initial-onset childhood systemic lupus erythematosus

**DOI:** 10.3389/fped.2023.1252594

**Published:** 2023-12-04

**Authors:** Lijun Jiang, Shufeng Zhi, Chenxi Wei, Zanhua Rong, Huifeng Zhang

**Affiliations:** Department of Pediatrics, The Second Hospital of Hebei Medical University, Shijiazhuang, China

**Keywords:** 25(OH)D, initial-onset childhood SLE, SLEDAI-2K, lupus nephritis, renal pathology

## Abstract

**Background:**

Vitamin D deficiency is common in patients with systemic lupus erythematosus (SLE) and may affect their disease activity and severity.

**Objective:**

This study aims to assess the vitamin D status in patients with initial-onset SLE during childhood and its association with the clinical and laboratory markers of disease activity.

**Method:**

This is a retrospective study that includes 168 patients with initial-onset SLE during childhood and 109 healthy children as controls. Clinical and laboratory data were recorded. The area under the curve (AUC) method was used to evaluate the efficacy of double-stranded deoxyribonucleic acid (dsDNA), lower 25(OH)D and complement 3 (C3) alone and in combination to diagnose the presence of renal damage in children with SLE.

**Result:**

Compared with the controls (25.53 ± 7.02 ng/ml), patients with initial-onset SLE during childhood have lower serum 25(OH)D levels (18.63 ± 5.32 ng/ml) (*P *< 0.05). Among patients with initial-onset SLE during childhood, SLEDAI-2K scores are significantly higher in the vitamin D insufficiency (median = 14.5) and vitamin D deficiency (median = 14.0) groups than in the vitamin D sufficiency group (median = 9.0) (*P *< 0.05). Patients with initial-onset SLE during childhood with lower 25(OH)D levels are more likely to have lupus nephritis (LN) and a higher SDI score (*P *< 0.05). Compared with patients with other types of LN (16.69 ± 3.90 ng/ml), patients with type V LN have lower levels of 25(OH)D (12.27 ± 3.53 ng/ml) (*P *< 0.05). The AUC was 0.803 when dsDNA antibody, 25(OH)D level and C3 were used in combination to diagnose LN in patients with SLE.

**Conclusion:**

Vitamin D deficiency and insufficiency are closely related to an increase in SLEDAI and SDI scores. Significant decrease in vitamin D level is a risk factor for LN.

## 1. Introduction

Systemic lupus erythematosus (SLE) is a systemic autoimmune disease that causes chronic inflammation and damage to multiple tissue types and organs, including the brain, joints, blood vessels, kidneys and skin (1). Lupus nephritis (LN) is a common complication of SLE and the main cause of death among SLE patients. It is estimated that 50%–60% of SLE patients will develop nephritis within 10 years from the onset of SLE and 20% of SLE patients will develop severe LN (2, 3). The prognosis of patients with early-onset LN is poor (4). The clinical manifestations of initial-onset SLE in childhood are more severe and the prognosis is worse. The pathogenesis of SLE is not fully understood. The current treatment for SLE includes the administration of hormones, immunosuppressants and biological agents but these drugs have relatively substantial side effects. Accordingly, new treatment methods are being explored.

Vitamin D is an important steroid hormone that has significant effects on bone health and on the cardiovascular system (5). Vitamin D has been shown to have potent immunomodulatory effects on both innate and adaptive immune systems (6, 7). Most vitamin D is produced by the body through sunlight, while only a small amount is obtained from diet. Vitamin D deficiency was found in many patients with autoimmune diseases (8–11). Studies showed that patients with SLE nephritis were at a higher risk of vitamin D deficiency than patients who did not have SLE (12).

In SLE, vitamin D deficiency is associated with increased disease activity and severity. Kamen et al. (13) found that SLE patients with low serum vitamin D levels had higher levels of autoantibodies and more frequent disease flare-ups than those with normal levels. Abou-Raya et al. (14) showed that vitamin D supplementation in SLE patients reduced disease activity and improved quality of life. Following a diagnosis, patients with SLE must avoid sunlight, which hinders the production of vitamin D (7). Considering that sun avoidance could affect the results of this study, the participants included in our investigation were patients with initial-onset SLE.

The vast majority of studies address adult SLE. Children's need for vitamin D is more critical during growth and development stages, but there is little research on the relationship between childhood SLE and vitamin D. There are fewer large-scale studies on vitamin D levels and clinical manifestations in paediatric patients with initial-onset SLE. Our centre collected the data of 168 children with initial-onset SLE from 2015 to 2022 and conducted a detailed analysis of clinical manifestations, laboratory tests, renal pathology and vitamin D levels.

This research reflects a retrospective study aiming to assess the vitamin D status in patients with initial-onset childhood SLE and its association with clinical and laboratory parameters of disease activity.

## 2. Materials and methods

### 2.1. Research participants

A total of 168 children aged <18 years with initial-onset SLE who were admitted to the Paediatrics Department of our hospital from February 2014 to May 2022 were included in this study. Concurrently, in the same period, healthy children (*n* = 109) in the outpatient department who matched the age and gender were selected as the control group. This study was approved by the ethics committee of our hospital. This study informed all the family members of the study participants by phone and obtained informed consent for their inclusion in the research.

The inclusion criteria for the research participants were as follows: (1) patients met the 1997 American College of Rheumatology (ACR) classification criteria for SLE (15) or the 2012 Systemic Lupus Erythematosus International Collaborating Clinics (SLICC) classification criteria for SLE (16); (2) patients younger than 18 years old; (3) patients diagnosed with SLE for the first time.

The exclusion criteria were as follows: (1) certain diseases that affect vitamin D metabolism (gastrointestinal surgery, liver metabolic diseases, tumours, etc.); (2) vitamin D supplementation by oral medication within the past 3 months; (3) corticosteroid treatment exceeding 10 mg/day within the past 3 months.

The diagnostic criteria for LN were the following. Children diagnosed with SLE were diagnosed with LN if they had any of the following renal involvement (17): (a) urine protein test results met any one of the following criteria—qualitative urine protein test positive 3 times within 1 week; 24-h urine protein >150 mg; urine protein/urine creatinine >0.2 mg/mg or urinary microalbumin higher than the normal reference value 3 times within 1 week; (b) centrifuged urine red blood cell count per high-power field of view >5; (c) abnormal glomerular and (or) renal tubule function; and (or) abnormal renal biopsy consistent with pathological changes caused by LN.

### 2.2. Renal pathological classification

The 2003 International Society of Nephrology (ISN)/Renal Pathology Society (RPS) classification of LN was adopted as the reference standard for the pathological classification of childhood LN (18). Lupus nephritis was divided into types I–VI based on different pathological manifestations.

### 2.3. SLE disease activity index (SLEDAI) and SLICC ACR-DI score (SDI) assessment

Disease activity was assessed using the SLE Disease Activity Index-2000 (SLEDAI-2K) (19). Using this index, disease activity was divided into mild (≤6 points), moderate (7–12 points) and severe (≥13 points) activity. Organ damage was assessed using the SDI (20).

### 2.4. Laboratory tests

Fasting venous blood (3–5 ml) was collected in the morning and sent to the medical experimental centre of the hospital for testing (conducted using an ADVIA Centaur XP automatic chemiluminescence immunoassay analyser). Laboratory examinations included routine blood tests, 24-h urine protein, erythrocyte sedimentation rate (ESR), liver function, renal function, complement 3 (C3), complement 4 (C4), antinuclear antibody, double-stranded deoxyribonucleic acid (dsDNA), serum calcium and serum phosphorus. Corrected calcium concentration was calculated using Payne's formula (21): when the serum albumin of the child was less than 40 g/L, corrected calcium concentration (mmol/L) = measured calcium concentration (mmol/L) + 0.02 × [40-serum albumin (g/L)]. The glomerular filtration rate (GFR) was estimated using the serum creatinine formula published by the Chronic Kidney Disease Epidemiology Collaboration (2009) (22).

### 2.5. Detection of serum 25(OH)D

Blood was collected between 6:00 and 7:00 in the morning, and the children were fasted from food and water overnight before the blood samples were collected. The detection of serum 25(OH)D was carried out using the chemiluminescence method (kit provided by Siemens Healthcare Diagnostics Inc., USA), and the analysis was conducted using an ADVIA Centaur XP automatic chemiluminescence immunoassay analyser.

### 2.6. Statistical analysis

All analyses were performed using SPSS 23.0 statistical software. Continuous variables were expressed as the mean ± standard deviation or median, and categorical variables were expressed as frequencies and percentages. Rates were compared between 2 or more groups using the chi-square test or Fisher's exact test. Continuous variables were compared among groups using the non-parametric Mann–Whitney *U*-test or Kruskal–Wallis test. To assess the correlation between two continuous variables, Pearson's correlation analysis was used for variables that conformed to a normal distribution and Spearman's correlation analysis for variables that did not. Receiver operating characteristic (ROC) curves and area under the ROC curve (AUC) were used to assess the probability of developing LN in patients and to assess the role of 25(OH)D in this model. A *P*-value less than 0.05 was considered statistically significant.

## 3. Results

### 3.1. Comparison of serum 25(OH)D levels in initial-onset childhood SLE patients and healthy controls

**Figure 1:**
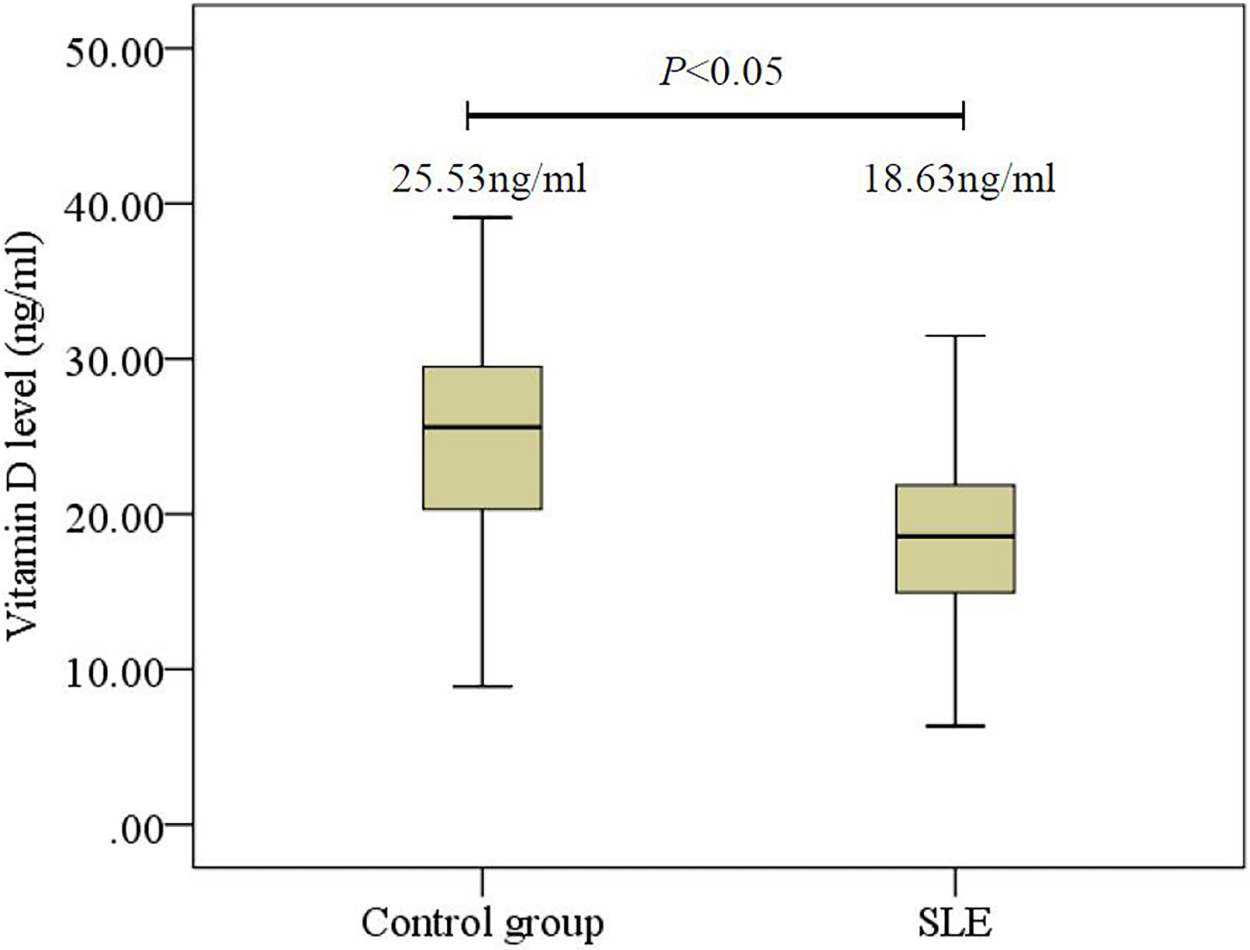
Comparison of 25(OH)D levels in initial-onset childhood patients and healthy controls. SLE, systemic lupus erythematosus.

In this study, there were 168 children with lupus (139 girls and 29 boys, aged 11.1 ± 2.4 years). For the healthy controls, there were 90 girls and 19 boys, aged 10.1 ± 2.4 years. The average serum 25(OH)D for children with SLE was 18.63 ± 5.32 ng/ml, and the average serum 25(OH)D for the healthy controls was 25.53 ± 7.02 ng/ml. The serum 25(OH)D for children with SLE was significantly lower than for the healthy controls (*P *< 0.05) (Figure 1).

### 3.2. Comparison of clinical and laboratory findings in patients with initial-onset SLE during childhood with different 25(OH)D levels

In this study, we compared the clinical manifestations and laboratory findings in three groups of children with lupus (Table 1). Patients with SLE were divided into tertiles according to their vitamin D level (23, 24): vitamin D deficiency [25(OH)D <12 ng/ml], vitamin D insufficiency [12 ng/ml ≤25(OH)D <20 ng/ml] and vitamin D sufficiency [25(OH)D ≥20 ng/ml] groups. The vitamin D level was negatively associated with the incidence of lupus nephritis (*P* < 0.001), serositis (*P* < 0.001), proteinuria (*P* < 0.001), 24-h urine protein (*P* < 0.001) and creatinine (*P* < 0.001). Compared with the vitamin D deficiency and D-insufficiency groups, the D-sufficiency group tended to have a higher level of serum albumin (*P* < 0.001) and estimated GFR (*P* < 0.001). The incidence of LN was higher in the vitamin D deficiency (71.4%) and vitamin D-insufficiency (68.4%) groups compared with the vitamin D-sufficiency group (27.7%) (*P* < 0.05).

**Table 1:** Demographic, clinical and laboratory characteristics of studied groups.

Characteristic	Total	25(OH)D <12 ng/ml	12 ng/ml ≤25(OH)D <20 ng/ml	25(OH)D ≥20 ng/ml	*P*
Female sex, *n*/*N* (%)	139/168 (82.7%)	17/19 (89.5%)	66/84 (78.6%)	56/65 (86.2%)	0.340
Age (years), mean ± SD	11.1 ± 2.4	11.6 ± 2.8	10.9 ± 2.6	11.1 ± 2.0	0.434
BMI (kg/m^2^), mean ± SD	18.06 ± 3.33	18.24 ± 3.45	18.00 ± 3.25	18.09 ± 3.44	0.948
Lupus nephritis, *n* (%)	91 (54.2%)	13 (68.4%)	60 (71.4%)	18 (27.7%)	<0.001
Arthritis, *n* (%)	38 (22.6%)	2 (10.5%)	18 (21.4%)	18 (27.7%)	0.313
Mucocutaneous, *n* (%)	86 (51.2%)	7 (36.8%)	42 (50.0%)	37 (56.9%)	0.304
Vasculitis, *n* (%)	2 (1.2%)	1 (5.3%)	1 (1.2%)	0 (0%)	0.222
Serositis, *n* (%)	20 (11.9%)	7 (36.8%)	9 (10.7%)	4 (6.2%)	0.001
Neurologic, *n* (%)	28 (16.7%)	4 (21.1%)	12 (14.8%)	12 (18.5%)	0.685
Pulmonary, *n* (%)	6 (31.6%)	19 (22.6%)	25 (24.3%)	7 (10.8%)	0.063
SLEDAI, median (range)	12.5 (3–37)	14 (3–36)	14.5 (3–37)	9 (3–31)	0.002
SDI ≥1, *n* (%)	52 (31.0%)	11 (57.9%)	34 (40.5%)	7 (10.8%)	<0.001
Leukopenia (<4 × 10^9^/L), *n* (%)	77 (45.8%)	7 (36.8%)	37 (44.0%)	33 (50.8%)	0.505
Aneamia (<110 g/L), *n* (%)	92 (54.8%)	10 (52.6%)	52 (61.9%)	30 (46.2%)	0.157
Thrombocytopenia (<100 × 10^9^/L), *n* (%)	54 (32.1%)	7 (36.8%)	27 (32.1%)	20 (30.8%)	0.883
Proteinuria, *n* (%)	88 (52.4%)	13 (68.4%)	59 (70.2%)	16 (24.6%)	<0.001
24-h urine protein (g), median (range)	0.22 (0.01–16.63)	3.04 (0.04–11.36)	0.69 (0.01–16.63)	0.08 (0.02–4.74)	<0.001
Creatinine (μmol/L), median (range)	41.0 (19.0–1,057.0)	45.0 (33.4–297.0)	43.2 (24.3–1,057.0)	38.0 (19.0–63.0)	<0.001
WBC count (×10^9^/L), mean ± SD	5.1 ± 3.5	6.0 ± 3.5	5.2 ± 3.8	4.8 ± 2.9	0.350
Neutrophil count (×10^9^/L), mean ± SD	3.2 ± 2.1	3.7 ± 2.4	3.3 ± 3.4	2.9 ± 2.1	0.520
Lymphocyte count (×10^9^/L), mean ± SD	1.5 ± 0.9	1.7 ± 1.1	1.5 ± 0.8	1.4 ± 0.9	0.625
CRP (mg/L), median (range)	1.20 (0.10–156.90)	1.98 (0.20–8.00)	1.10 (0.10–156.90)	1.20 (0.10–29.70)	0.864
Hemoglobin (g/L), mean ± SD	102.9 ± 24.5	99.2 ± 25.9	100.1 ± 24.3	107.5 ± 23.9	0.149
Platelet count (×10^9^/L), mean ± SD	155.9 ± 101.4	165.0 ± 109.4	144.2 ± 88.3	168.5 ± 113.9	0.322
Serum albumin (g/L), mean ± SD	33.8 ± 8.8	26.7 ± 11.6	31.6 ± 8.3	38.7 ± 5.5	<0.001
eGFR (ml/min/1.73 m^2^), mean ± SD	144.16 ± 41.11	123.35 ± 46.75	136.04 ± 48.6	160.75 ± 15.58	<0.001
ESR (mm/h), median (range)	38 (1–140)	61 (5–100)	37 (1–140)	36 (1–123)	0.162
C3 (g/L), mean ± SD	0.41 ± 0.22	0.42 ± 0.21	0.35 ± 0.21	0.48 ± 0.22	<0.001
C4 (g/L), mean ± SD	0.06 ± 0.05	0.07 ± 0.04	0.06 ± 0.05	0.07 ± 0.05	0.015
Positive anti-dsDNA, *n* (%)	118 (70.2%)	11 (57.9%)	64 (76.2%)	43 (66.2%)	0.189
Serum calcium (mmol/L), mean ± SD	2.09 ± 0.19	1.97 ± 0.24	2.05 ± 0.18	2.19 ± 0.14	<0.001
Corrected serum calcium (mmol/L), mean ± SD	2.24 ± 0.11	2.24 ± 0.11	2.23 ± 0.11	2.25 ± 0.10	0.538
Serum phosphorus (mmol/L), mean ± SD	1.48 ± 0.39	1.43 ± 0.33	1.51 ± 0.45	1.45 ± 0.31	0.738

25(OH)D, 25 hydroxyvitamin D; BMI, body mass index; SLEDAI, systemic lupus erythematosus disease activity index; SDI, SLICC ACR-DI score; WBC, white blood cell; CRP, C-reactive protein; eGFR, estimated glomerular filtration rate; ESR, erythrocyte sedimentation rate; C3, complement 3; C4, complement 4; ds-DNA, double-stranded deoxyribonucleic acid.

Among the three groups, the vitamin D-deficient group had the highest percentage of plasmacytosis (36.8%) and SDI ≥1 (57.9%) (*P *< 0.05). The SLEDAI-2K score was higher in the vitamin D-insufficiency group (median = 14.5) than in the vitamin D-sufficiency group (median = 9) (*P *< 0.05), indicating a higher disease activity in lupus. Compared with the vitamin D sufficiency group (C3 0.48 ± .022 g/L, C4 0.07 ± 0.05 g/L), children with lupus in the vitamin D-insufficiency group had lower levels of C3 and C4 (C3 0.35 ± 0.21 g/L, C4 0.06 ± 0.05 g/L) (*P *< 0.05). Differences related to neurological, pulmonary and mucocutaneous involvement, as well as arthritis and serum calcium among the three groups were not statistically significant (*P *> 0.05) (Table 1).

### 3.3. Comparison of the clinical data of patients with initial-onset SLE during childhood with different disease activity

Based on the SLEDAI-2K score, patients with initial-onset SLE during childhood were divided into 3 groups: SLEDAI = 0–6, low disease activity group; SLEDAI = 7–12, moderate disease activity group; and SLEDAI ≥13, high disease activity group. There were significant differences in 25(OH)D levels among the 3 groups, with the high disease activity group having lower 25(OH)D levels compared with the other 2 groups. There was no significant difference in 25(OH)D levels between the moderate disease activity and low disease activity groups. Serum calcium levels differed among the three groups; the higher the disease activity in SLE patients, the lower the serum calcium levels were. However, there was no significant difference among the three groups in serum calcium levels corrected for serum albumin. There were no statistically significant differences in sex, age or body mass index (BMI) among the different disease activity groups (Table 2).

**Table 2:** Comparison of the clinical data of initial-onset childhood lupus patients with different disease activities.

	SLEDAI (0–6) (*n* = 33)	SLEDAI (7–12) (*n* = 51)	SLEDAI (≥13) (*n* = 84)	*P*
Female sex, *n*/*N* (%)	24/33 (72.7%)	46/51 (90.2%)	69/84 (82.1%)	0.115
Age (years), mean ± SD	11.1 ± 2.0	11.3 ± 2.2	10.9 ± 2.6	0.914
BMI (kg/m^2^), mean ± SD	17.37 ± 2.85	17.82 ± 3.30	18.48 ± 3.48	0.273
25(OH)D (ng/ml), mean ± SD	19.53 ± 4.80	20.29 ± 6.16	17.26 ± 4.61	0.001
Serum calcium (mmol/L), mean ± SD	2.25 ± 0.15	2.13 ± 0.14	2.01 ± 0.19	<0.001
Corrected serum calcium (mmol/L), mean ± SD	2.28 ± 0.13	2.23 ± 0.08	2.23 ± 0.11	0.053
Serum phosphorus (mmol/L), mean ± SD	1.53 ± 0.26	1.41 ± 0.14	1.50 ± 0.47	0.183

SLEDAI, systemic lupus erythematosus disease activity index; BMI, body mass index; 25(OH)D, 25 hydroxyvitamin D.

### 3.4. Comparisons of the clinical data of children with and without LN

Children with SLE were divided into 2 groups based on renal involvement as the LN and non-LN groups. The 25(OH)D level in the LN group was 17.16 ± 4.85 ng/ml, and the 25(OH)D level in the non-LN group was 20.37 ± 5.35 ng/ml. Compared with the non-LN group (2.20 ± 0.14 mmol/L), the LN group had a lower serum calcium level (2.00 ± 0.18 mmol/L); however, serum calcium after serum albumin correction was not significantly different. The ESR was faster in the LN group (median, 47 mm/h) compared with the non-LN group (median, 33 mm/h). The levels of C3 and C4 in the LN group were lower compared with the non-LN group, and the proportion of children with positive dsDNA antibodies was higher in the LN compared with the non-LN group. There were no significant differences in sex, age and BMI between the LN and non-LN groups (Table 3).

**Table 3:** Comparison of the clinical data of LN and non-LN patients.

	Non-LN (*n* = 77)	LN (*n* = 91)	*P*
Female sex, *n*/*N* (%)	64/77 (83.1%)	75/91 (82.4%)	0.905
Age (years), mean ± SD	11.2 ± 2.1	11.0 ± 2.7	0.626
BMI (kg/m^2^), mean ± SD	17.81 ± 3.22	18.27 ± 3.41	0.290
25(OH)D (ng/ml), mean ± SD	20.37 ± 5.35	17.16 ± 4.85	<0.001
eGFR (ml/min/1.73 m^2^), mean ± SD	158.94 ± 14.13	131.66 ± 51.20	0.001
24-h urinary protein (g), median (range)	0.07 (0.01–0.14)	1.5 (0.05–16.63)	<0.001
Serum calcium (mmol/L), mean ± SD	2.20 ± 0.14	2.00 ± 0.18	<0.001
Corrected serum calcium (mmol/L), mean ± SD	2.25 ± 0.11	2.23 ± 0.11	0.533
Serum phosphorus (mmol/L), mean ± SD	1.45 ± 0.27	1.49 ± 0.46	0.854
ESR, median (range)	33 (1–123)	47 (5–140)	0.014
C3, mean ± SD	0.51 ± 0.23	0.32 ± 0.18	<0.001
C4, mean ± SD	0.08 ± 0.05	0.05 ± 0.04	<0.001
Positive anti-dsDNA, *n* (%)	46/77 (59.7%)	72/91 (79.1%)	0.006

LN, lupus nephritis; BMI, body mass index; 25(OH)D, 25 hydroxyvitamin D; eGFR, estimated glomerular filtration rate; ESR, erythrocyte sedimentation rate; C3, complement 3; C4, complement 4; ds-DNA, double-stranded deoxyribonucleic acid.

**Figure 2:**
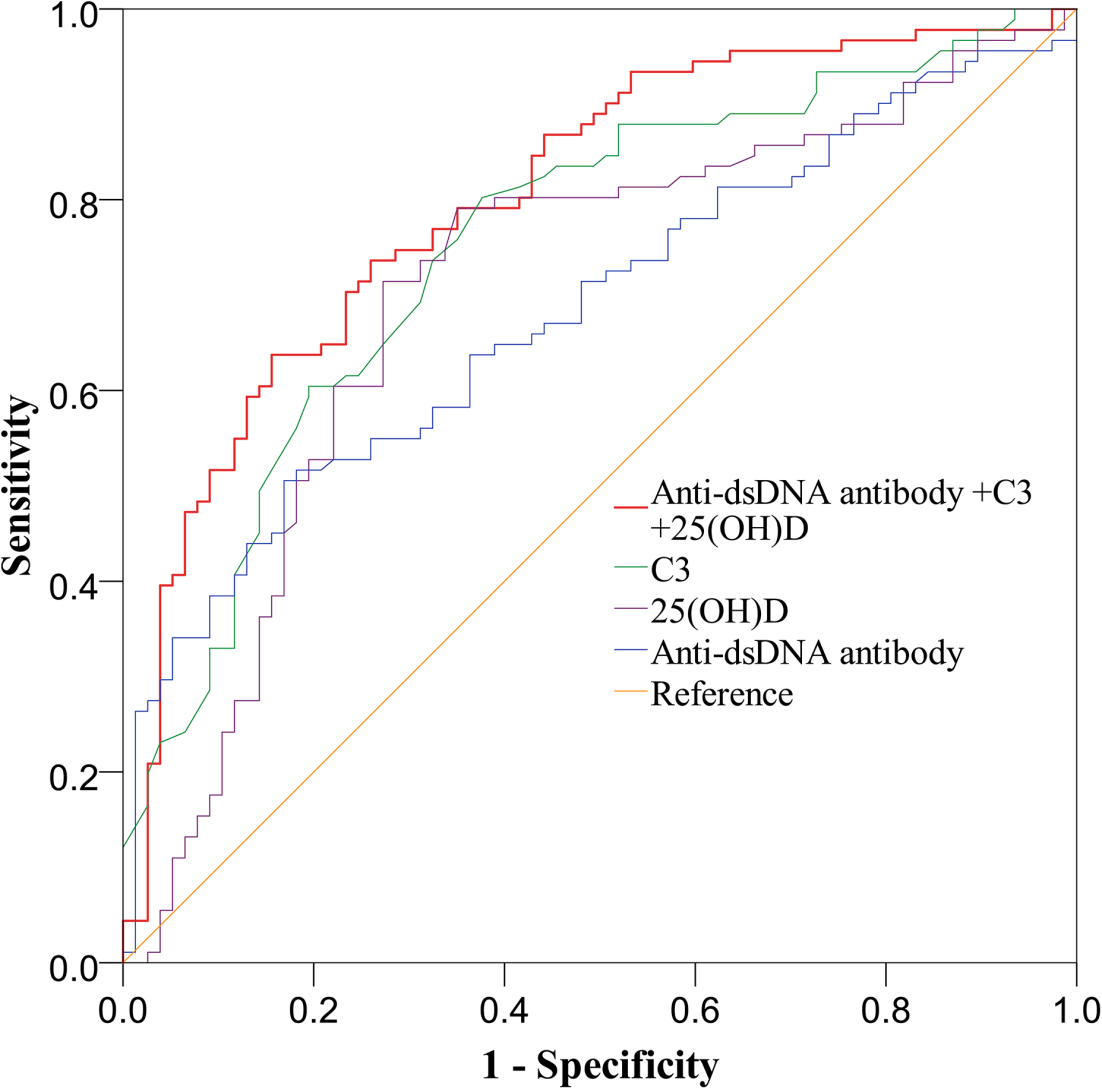
Receiving operating characteristics (ROC) curve analysis for identifying variables predicting the diagnosis of LN in children with SLE. C3, complement 3; 25(OH)D, 25 hydroxyvitamin D; ds-DNA, double-stranded deoxyribonucleic acid.

In this study, ROC curves were drawn to explore the risk factors for LN. The results indicated that dsDNA antibody was a risk factor for LN (AUC, 0.683). The AUCs for the 25(OH)D level and C3 in LN were 0.705 and 0.754, respectively. When anti-dsDNA antibodies, 25(OH)D level and C3 were combined to predict LN, the AUC was 0.803 (Figure 2). Decreased 25(OH)D may be one of the risk factors for LN.

### 3.5. Differences in clinical parameters between the SDI = 0 and SDI ≥1 groups

The children with SLE were divided into 2 groups based on SDI score: SDI = 0 group and SDI ≥1 group. There was no significant difference in the sex ratio or age between the 2 groups. The BMI of children with SLE in the SDI ≥1 group (19.14 ± 3.72 kg/m^2^) was higher compared with the SDI = 0 group (17.58 ± 3.03 kg/m^2^). The calculated GFR for children with SLE in the SDI ≥1 group (113.91 ± 57.63 ml/min/1.73 m^2^) was significantly lower than for children in the SDI = 0 group (157.72 ± 19.54 ml/min/1.73 m^2^). The 24-h urine protein level (median = 3.84 g) in the SDI ≥1 group was significantly higher than in the SDI = 0 group (median = 0.09 g). The level of C3 in the SDI ≥1 group (0.34 ± 0.18 g/L) was lower than in the SDI = 0 group (0.44 ± 0.23 g/L). Children in the SDI ≥1 group had significantly lower levels of 25(OH)D and serum calcium and higher levels of serum phosphorus. Calcium concentrations adjusted for albumin were not significantly different between the 2 groups (Table 4).

**Table 4:** Comparison of clinical indicators between the SDI = 0 group and the SDI ≥ 1 group.

	SDI = 0 (*n* = 116)	SDI ≥ 1 (*n* = 52)	*P*
Female sex, *n*/*N* (%)	96/116 (82.8%)	43/52 (82.7%)	0.992
Age (years), mean ± SD	10.99 ± 2.22	11.21 ± 2.75	0.273
BMI (kg/m^2^), mean ± SD	17.58 ± 3.03	19.14 ± 3.72	0.007
25(OH)D (ng/ml), mean ± SD	19.87 ± 5.28	15.86 ± 4.30	<0.001
eGFR (ml/min/1.73 m^2^), mean ± SD	157.72 ± 19.54	113.91 ± 57.63	<0.001
24-h urinary protein (g), median (range)	0.09 (0.01–4.03)	3.84 (0.04–16.63)	<0.001
Serum calcium (mmol/L), mean ± SD	2.16 ± 0.15	1.95 ± 0.20	<0.001
Corrected serum calcium (mmol/L), mean ± SD	2.24 ± 0.10	2.22 ± 0.11	0.681
Serum phosphorus (mmol/L), mean ± SD	1.42 ± 0.30	1.60 ± 0.51	0.026
ESR (median, range)	36 (1–140)	53 (5–140)	0.136
C3, mean ± SD	0.44 ± 0.23	0.34 ± 0.18	0.017
C4, mean ± SD	0.07 ± 0.05	0.06 ± 0.04	0.993
Positive anti-dsDNA, *n* (%)	77/116 (66.4%)	41/52 (78.8%)	0.102

SDI, SLICC ACR-DI score; BMI, body mass index; 25(OH)D, 25 hydroxyvitamin D; eGFR, estimated glomerular filtration rate; ESR, erythrocyte sedimentation rate; C3, complement 3; C4, complement 4; ds-DNA, double-stranded deoxyribonucleic acid.

### 3.6. Correlation between 25(OH)D levels and clinical laboratory parameters

The 25(OH)D levels were positively correlated with GFR (*R* = 0.237, *P *= 0.002) and C3 (*R* = 0.233, *P *= 0.002). The 25(OH)D levels were negatively correlated with 24-h urinary protein (*R* = −0.384, *P *< 0.001) and SLEDAI (*R* = −0.244, *P *= 0.001) scores (Table 5).

**Table 5:** Correlations between 25(OH)D levels and clinical laboratory indicators.

	R (spearman correlation)	*P*
eGFR	0.237	0.002
24-h urinary protein	−0.384	<0.001
C3	0.233	0.002
C4	0.043	0.582
ESR	−0.137	0.076
SLEDAI	−0.244	0.001

eGFR, estimated glomerular filtration rate; C3, complement 3; C4, complement 4; ESR, erythrocyte sedimentation rate; SLEDAI, systemic lupus erythematosus disease activity index.

### 3.7. Serum 25(OH)D levels in different types of LN

Of the 91 children with LN, 46 cases underwent renal biopsy. There were 9 cases of type II, 11 of type III, 20 of type IV and 6 cases of type V LN. The 25(OH)D level in children with type II LN was the highest (18.07 ± 4.50 ng/ml), followed by that in children with type III (16.45 ± 4.37 ng/ml), type IV (16.19 ± 3.59 ng/ml) and type V LN (12.27 ± 3.53 ng/ml). The differences in 25(OH)D levels between children with SLE with different renal pathology types were not significant (*P *= 0.073).

Different from other types of LN, the light microscopy pathological changes of type V LN were mainly diffuse thickening of the basement membrane and the formation of spike-like structures manifesting as membranous nephropathy. In this study, the 46 children with LN were divided into 2 groups based on renal pathology, that is, type V and non-type V LN. The 25(OH)D level in the type V LN group was significantly lower than in the non-type V LN group (*P *= 0.016) (Table 6).

**Table 6:** The 25(OH)D levels in children with type V LN and non-type V LN.

	Total	Non-type V LN (*n* = 40)	Type V LN (*n* = 6)	*P*
25(OH)D (ng/ml), mean ± SD	16.11 ± 4.17	16.69 ± 3.90	12.27 ± 3.53	0.016

LN, lupus nephritis; 25(OH)D, 25 hydroxyvitamin D.

## 4. Discussion

To our knowledge, this is the first study to investigate the relationship between serum 25(OH)D levels, disease activity and renal involvement in patients with initial-onset SLE during childhood from a single centre in China. Our study presents several novel findings that add new insight into the existing literature on this topic. First, we found that vitamin D deficiency and insufficiency were common in initial-onset childhood patients with SLE and were associated with higher disease activity and lower C3 levels. Second, we found that a low vitamin D level was a risk factor for LN and correlated with 24-h urinary protein and GFR. Third, we found that vitamin D levels differed among different types of LN, with type V LN reflecting the lowest vitamin D level.

Our findings are consistent with existing studies that reported lower vitamin D levels in patients with SLE compared to healthy controls, both in adults (25–29) and in children. However, our study is unique in that we included only patients with initial-onset SLE during childhood who had not received any treatment or had not been sun-avoidant before the study, thus eliminating the confounding effects of these factors on vitamin D status. Moreover, we compared the vitamin D levels of patients with SLE with those of healthy children who matched the age and gender of the patients with SLE, which increased the validity of our results.

Our study also confirmed the negative association between vitamin D levels and SLEDAI-2K scores reported in several studies in adult SLE patients (30–32). A systematic review and meta-analysis concluded that 25(OH)D levels were negatively associated with SLEDAI-2K scores (33). However, few studies have examined this association in paediatric SLE patients. One study from Brazil found that vitamin D supplementation reduced disease activity in SLE onset for juvenile patients (34), while another study from Iran found no significant effect of vitamin D supplementation on disease activity in patients with SLE. The discrepancy between these studies may be due to differences in the baseline vitamin D levels, disease activity scores, sample sizes and supplementation doses and durations (35). Our study suggests that vitamin D deficiency and insufficiency may contribute to the increase of disease activity in patients with childhood initial-onset SLE and that vitamin D supplementation may have potential benefits for this population.

Our study also revealed a significant association between vitamin D levels and renal involvement in patients with childhood initial-onset SLE. We found that children with LN had lower serum 25(OH)D levels than those without LN and that a low vitamin D level was a risk factor for LN. We also found that 25(OH)D levels were positively correlated with GFR and negatively correlated with 24-h urinary protein, indicating that vitamin D deficiency and insufficiency may impair renal function and increase proteinuria in patients with SLE. Moreover, we found that 25(OH)D levels differed among different renal pathology types, with type V LN having the lowest vitamin D level. These findings are consistent with some other studies on adult patients with SLE (36, 37) but inconsistent with others (38). The reasons for these inconsistencies may be related to the differences in diagnostic criteria, classification methods, sample sizes and the ethnic backgrounds of patients with SLE. Our study suggests that vitamin D deficiency and insufficiency may play a role in the pathogenesis and progression of LN in patients with childhood initial-onset SLE and that vitamin D supplementation may have protective effects on renal function and structure.

The possible mechanisms by which vitamin D may influence the development and progression of SLE and LN are not yet fully understood, but several hypotheses have been proposed. Vitamin D may modulate the immune system by affecting various immune cells, such as T cells, B cells, dendritic cells and macrophages. Vitamin D may suppress the production of pro-inflammatory cytokines, such as interleukin-6 (IL-6), tumour necrosis factor-alpha, interferon-gamma and IL-17, which are involved in the pathogenesis of SLE. Vitamin D may also induce the expression of anti-inflammatory cytokines, such as IL-10 and transforming growth factor-beta, which have regulatory effects on immune tolerance. Vitamin D may also inhibit the activation and differentiation of B cells and reduce the production of autoantibodies, such as anti-dsDNA antibodies. Vitamin D may also protect against LN by reducing renal inflammation, fibrosis and oxidative stress. Vitamin D may also regulate the renin-angiotensin-aldosterone system, which is implicated in the development of hypertension and proteinuria in LN. Vitamin D may also modulate the expression of vitamin D receptors and vitamin D binding proteins in the kidneys, which may affect the local availability and activity of vitamin D (38–42).

The limitations of this study include its retrospective design, the single-centre setting, the small sample size and the lack of a control group of healthy children with matched vitamin D levels. The strengths of this study include the inclusion of only patients with SLE where the initial onset occurred during childhood, the comprehensive assessment of clinical and laboratory markers of disease activity and renal involvement and the comparison of vitamin D levels among different types of LN.

Based on our findings, we suggest that vitamin D supplementation may have potential benefits for patients with SLE where the initial onset occurred during childhood, especially those with LN or high disease activity. Vitamin D supplementation may improve their immune function, reduce inflammation, protect kidney function and enhance patients' quality of life. However, more studies are needed to determine the optimal dose, duration and frequency of vitamin D supplementation for this population. Moreover, it would be interesting to explore whether vitamin D supplementation could prevent or delay the onset of SLE in children who are at risk of developing the disease, such as those with a family history or genetic predisposition.

## 5. Conclusion

In conclusion, the prevalence of vitamin D deficiency and insufficiency in Chinese children with SLE is high. Vitamin D deficiency and insufficiency are strongly associated with elevated SLEDAI and SDI scores. A decreased 25(OH)D level is a risk factor for LN. The levels of 25(OH)D vary among different types of LN, with the lowest 25(OH)D level observed for type V LN. The mechanism of action of vitamin D in SLE requires further study.

## Data Availability

The original contributions presented in the study are included in the article/Supplementary Material, further inquiries can be directed to the corresponding author.
